# TUSC2 downregulates PD-L1 expression in non-small cell lung cancer (NSCLC)

**DOI:** 10.18632/oncotarget.22581

**Published:** 2017-11-21

**Authors:** Xiaobo Cao, Yang Zhao, Jing Wang, Bingbing Dai, Emanuela Gentile, Jing Lin, Xingxiang Pu, Lin Ji, Shuhong Wu, Ismail Meraz, Mourad Majidi, Jack A. Roth

**Affiliations:** ^1^ Department of Thoracic and Cardiovascular Surgery, Section of Thoracic Molecular Oncology, The University of Texas MD Anderson Cancer Center, Houston, Texas, USA; ^2^ Department of Bioinformatics and Computational Biology, The University of Texas MD Anderson Cancer Center, Houston, Texas, USA; ^3^ Department of Surgical Oncology, The University of Texas MD Anderson Cancer Center, Houston, Texas, USA; ^4^ Department of Thoracic Medical Oncology, Hunan Cancer Hospital, Changsha, China

**Keywords:** PD-L1, TUSC2, mTOR, protein translation, lung cancer

## Abstract

Expression of the *TUSC2* tumor-suppressor gene in TUSC2-deficient NSCLC cells decreased PD-L1 expression and inhibited mTOR activity. Overexpressing TUSC2 or treatment with rapamycin resulted in similar inhibition of PD-L1 expression. Both TUSC2 and rapamycin decreased p70 and SK6 phosphorylation, suggesting that TUSC2 and rapamycin share the same mTOR target. Microarray mRNA expression analysis using TUSC2-inducible H1299 showed that genes that negatively regulate the mTOR pathway were significantly upregulated by TUSC2 compared with control. The presence of IFN-γ significantly increased PD-L1 expression in lung cancer cell lines, but overexpressing TUSC2 in these cell lines prevented PD-L1 from increasing in the presence of IFN-γ. Taken together, these findings show that TUSC2 can decrease PD-L1 expression in lung cancer cells. This ability to modify the tumor microenvironment suggests that TUSC2 could be added to checkpoint inhibitors to improve the treatment of lung cancer.

## INTRODUCTION

Non-small cell lung carcinoma (NSCLC) is one of the most aggressive and devastating malignancies. Treatment of NSCLC with conventional cisplatin-based therapies has had limited success. Median overall survival with these therapies is about 10 months [1]. Various small-molecule drugs have been used to target specific signaling pathways in lung cancer. Tyrosine kinase inhibitors, particularly inhibitors that target the epidermal growth factor receptor pathway, have promising therapeutic value for the treatment of lung cancer [2]. Unfortunately, resistance to tyrosine kinase inhibitors is often acquired, leading to tumor progression.

As downstream kinases, AKT/mTOR kinases are important regulators of multiple cellular processes, including metabolism, proliferation, and protein synthesis, as well as programmed cell death [3]. Because deregulated activation of AKT/mTOR occurs in 70% of cases of NSCLC, over-activated AKT/mTOR is a relevant therapeutic target in lung cancer as this pathway serves as the convergence for many growth stimuli, including cellular transformation, promotion of tumor invasion and angiogenesis, and resistance to chemotherapy and radiation therapy. Clinical evaluations of AKT/mTOR kinase inhibitors have shown some promising results [3].

Overexpression of PD-L1, a downstream target of AKT/mTOR, has been observed in many solid tumor types, including NSCLC [4]. PD-1 or PD-L1 monoclonal antibodies have been tested in clinical trials for the treatment of NSCLCs, with response rates of 10% to 30%. Although the significance of PD-L1 expression as a biomarker in lung cancer is controversial, PD-L1 expression should be considered in the context of an immune evasion environment created by cancer cells. An immunosuppressive microenvironment is a complex and dynamic state involving various molecules and cells, which originates from constitutively altered signals within cancer cells. These signals also regulate proliferation or metastasis and constitute oncogenic signals, as evidenced by STAT3, AKT/mTOR, or mutated BRAF [5, 6]. Extensive study has shown that PD-L1 expression is regulated at both the transcriptional level controlled by JAK/STATs activity [7] and the translational level controlled by mTOR activity [8]. The signal antagonists targeting these regulators have also been reported to contribute to PD-L1 reduction.

We previously reported that restoration of *TUSC2,* a tumor suppressor gene in the 3p21.3 region that has been extensively characterized in 3p21.3-deficient NSCLC cells suppressed tumor growth by induction of apoptosis and alteration of cell kinetics *in vitro* and *in vivo* through Apaf-1 [9]. In addition, results from our previous studies showed that TUSC2 inhibited the function of protein tyrosine kinases, including epidermal growth factor receptor, platelet-derived growth factor receptor, AKT, c-Abl, c-Kit, and mTOR. Restoration of TUSC2 expression in tumor cells significantly inhibited tumor growth and progression in mouse models. TUSC2 overexpression in mesothelioma was observed to decrease PD-L1 expression and elevate interleukin-15 expression and was associated with an immunologic response [10]. These findings led to phase I [11] and II clinical trials that showed that TUSC2 nanoparticle-based systemic gene therapy administered intravenously in lung cancer patients was safe and had antitumor activity.

Therefore, we hypothesized that TUSC2 is involved in the regulation of PD-L1 signaling and the immune response in the tumor microenvironment. In the current study, we examined the role of TUSC2 in regulating PD-L1 expression in NSCLC and showed that TUSC2 can mediate PD-L1 downregulation potentially enhancing the efficacy of the antitumor immune response.

## RESULTS

### TUSC2 reduction of PD-L1 expression in NSCLC cell lines is associated with reduced mTOR activity

**Figure 1:**
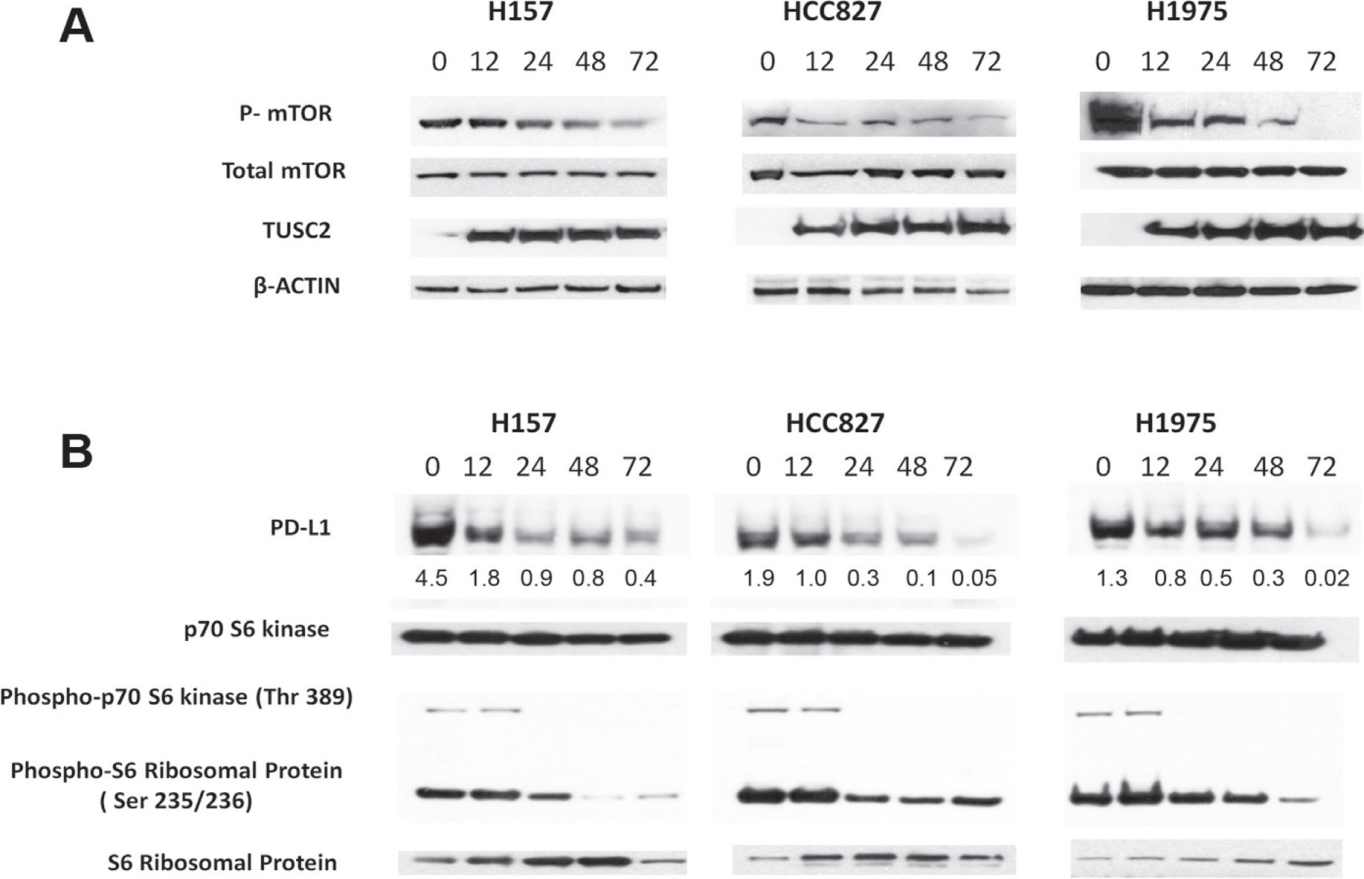
Transfection with TUSC2 reduces PD-L1 expression in non-small cell lung cancer H157, H1975, and HCC827 cells were transiently transfected with 4 μg of TUSC2 cDNA for 48 hours and the cells were then harvested and lysed. There were no significant toxicity observed. (**A**) Phospho-mTOR, mTOR, TUSC2, and β-actin control signals were detected using Western blot analysis. (**B**) Phospho-S6 ribosomal protein and phospho-p70 S6 kinase, as well as PD-L1, were detected by Western blot analysis. No change was observed in the S6 ribosomal protein, p70 S6 kinase protein. Proteins were quantified using the UN-SCAN-IT automatic digitizing system. Those intensities of the signals of PD-L1 are relative to β-actin.

Treatment with TUSC2 alone was shown to significantly reduce mTOR activity *in vitro* and *in vivo* [12, 13]. In some cell lines, mTOR kinase activity was inhibited by 70% after TUSC2 transfection alone. *In vivo*, TUSC2 systemic delivery alone led to a significant decrease in mTOR phosphorylation, as assessed by immunohistochemical staining using the phospho-mTOR antibody. Baseline expressions of PD-L1s and mTOR activities in sixteen lung cancer cell lines were illustrated in Supplementary Figure 1. The PD-L1 expressions were positively correlated to mTOR activities, demonstrated by S6 ribosomal protein phosphorylation. To evaluate whether the TUSC2 expression can reduce mTOR phosphorylation in cell lines in which PD-L1 is highly expressed, we transfected HCC827, H1975, and H157 cells with TUSC2 nanoparticles. We evaluated mTOR phosphorylation using Western blot analysis with an anti-phospho-mTOR antibody. We found overexpressing TUSC2 significantly reduced mTOR phosphorylation (Figure 1A).

To determine whether TUSC2 expression inhibits active PI3K/AKT/mTOR signaling and represses PD-L1, we treated human NSCLC cell lines in which PD-L1 was highly expressed with TUSC2 nanoparticles at multiple time points. Transfection with TUSC2 inhibited mTOR activity (Figure 1B) and decreased PD-L1 expression (Figure 1B) in a time-dependent manner. In addition, PD-L1 expression after TUSC2 delivery was correlated with a decrease of phosphorylated p70 S6 kinase and S6 ribosomal proteins, which are the downstream targets of mTOR (Figure 1B). Taken together, these results suggest that TUSC2 reduces PD-L1 expression in NSCLC in association with a reduction in mTOR activity.

### Exposure to the mTOR inhibitor rapamycin reduced PD-L1 expression in NSCLC cell lines

**Figure 2:**
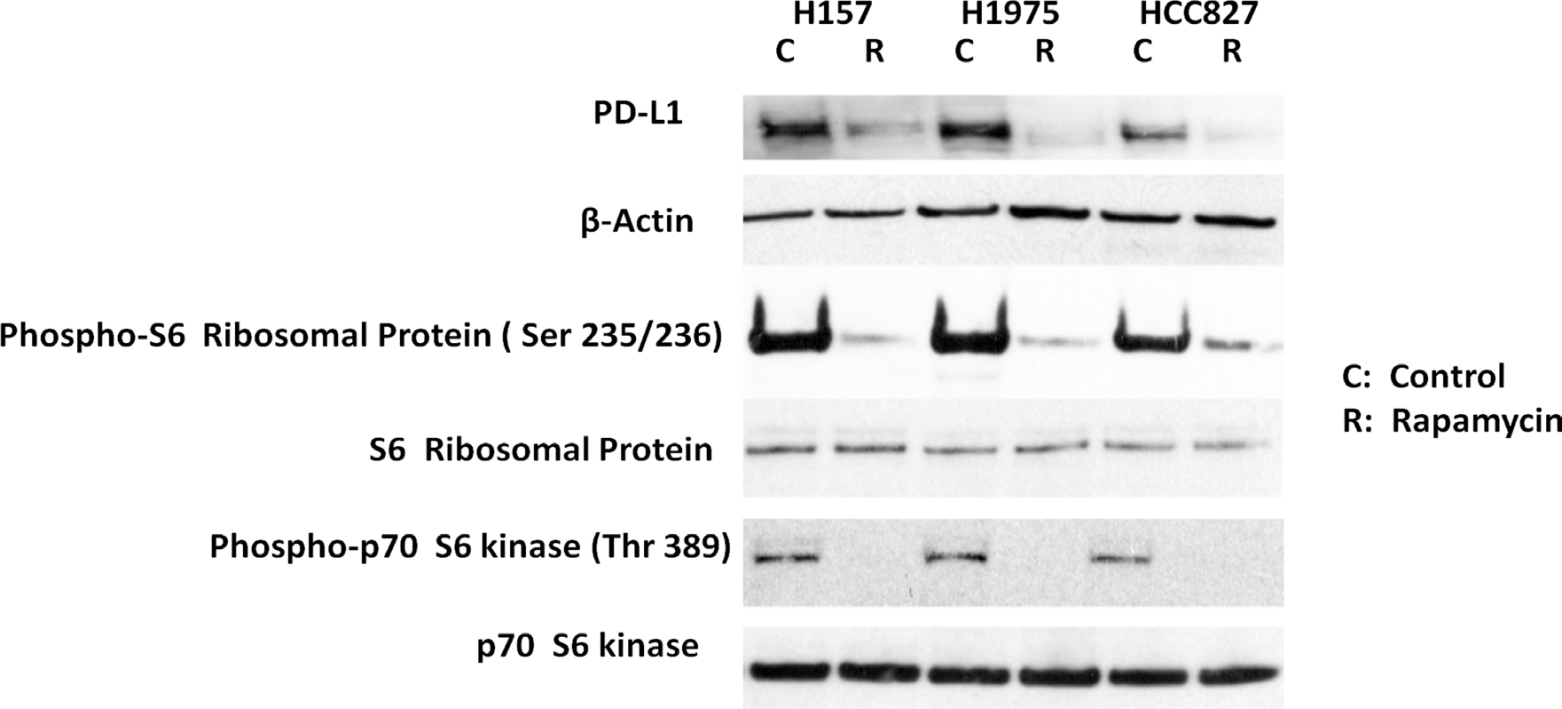
Exposure to the mTOR inhibitor rapamycin reduced PD-L1 expression in non-small cell lung cancer cell lines H157, H1975, and HCC827 cells were treated for 24 hours with 100 nmol/L rapamycin. These were no cytotoxicity found. Western blot analysis showed that phospho-S6 ribosomal protein and phospho-p70 S6 kinase, as well as PD-L1, were decreased. No change was observed in levels of S6 ribosomal protein, p70 S6 kinase protein, or β-actin (used to normalize for protein loading).

To further explore the relationship between PD-L1 and mTOR activity in lung cancer cells, we treated three NSCLC cell lines in which PD-L1 is highly expressed—H157, H1975, and HCC827 [14]—with rapamycin for 24 hours. Exposure to rapamycin decreased PD-L1 expression in these cell lines, as shown by Western blot analysis (Figure 2). In addition, we observed that rapamycin exposure significantly reduced phosphorylation of p70 S6 kinase and S6 ribosomal protein. These results indicate that PD-L1 expression is sensitive to mTOR inhibition, suggesting that decreasing mTOR activity can result in decreased PD-L1 expression.

### TUSC2 inhibits mTOR function through multiple proteins in NSCLC cell lines

**Figure 3:**
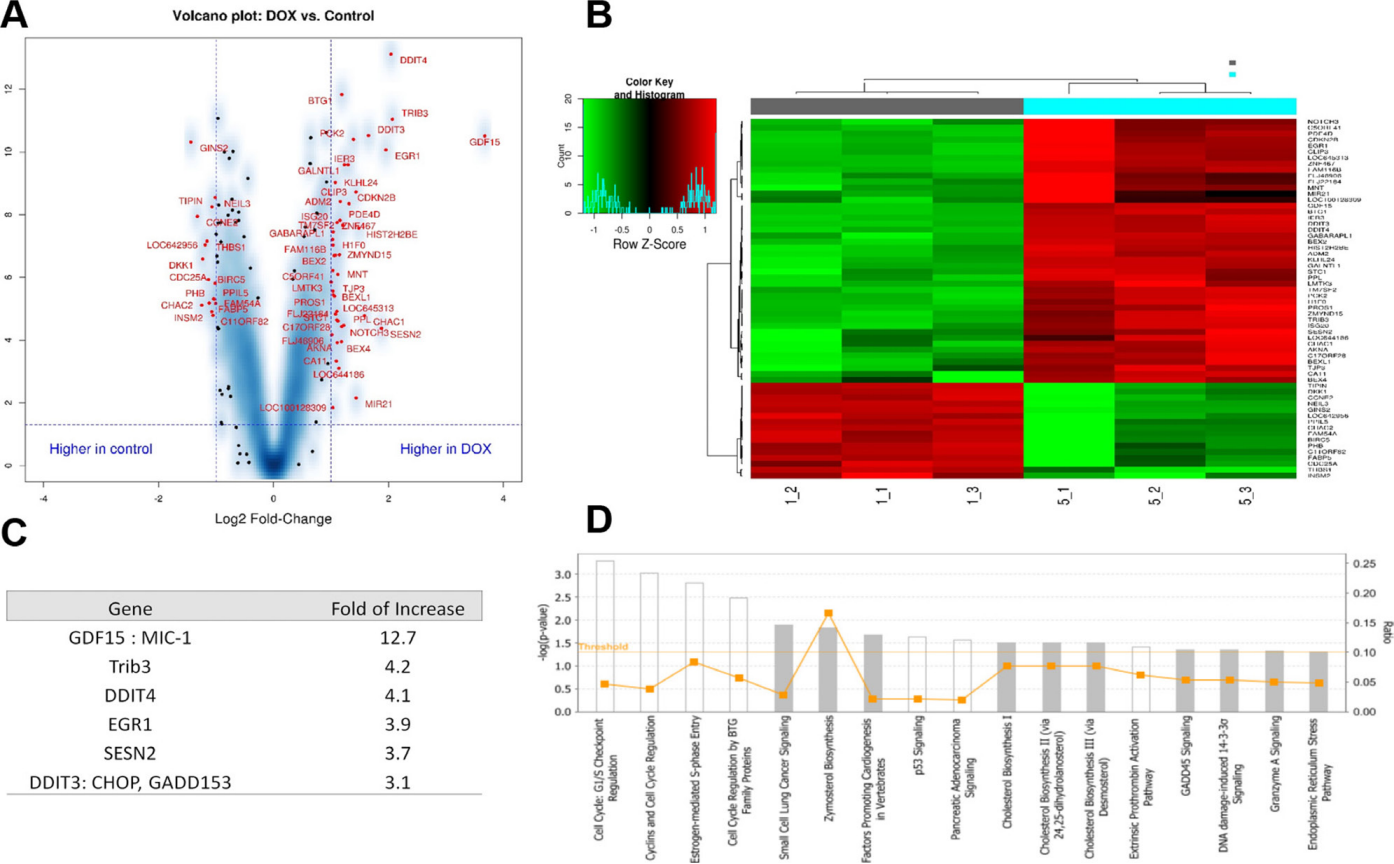
TUSC2 inhibits mTOR function through multiple proteins in non-small cell lung cancer cell lines The Illumina array human HT-12V4 expression bead chip platform was used across the Tet-inducible TUSC2 H1299 clone treated with doxycycline (DOX) or control. Each experiment was done in triplicates. One-way ANOVA was used to identify the differentially expressed genes. Tukey honest significant difference tests were used for the *post hoc* pairwise comparisons. (**A**) Volcano plot: genes marked in red with false discovery rate of 0.002 and fold changes ≥2 or ≤–2 were selected. (**B**) Heatmap: genes with false discovery rate of 0.002 and fold changes ≥2 or ≤–2 were considered significant genes. Control: gray; DOX: blue. (**C**) List of top genes significantly upregulated after treatment with DOX. (**D**) Ingenuity pathway analysis based on statistical significance and strength of association with extracts revealed the highest positive z-score for cell cycle arrest response, which is a downstream response to mTOR inhibition.

Upon observing that transfection with TUSC2 inhibited PD-L1 expression via mTOR inhibition, we used the mTOR pull-down assay to determine whether mTOR had any direct interaction with TUSC2. The negative result led us to suspect that the TUSC2 protein indirectly regulates mTOR activity. To identify differentially expressed genes and specific pathways involved in TUSC2-induced mTOR inhibition, we used the Illumina human HT-12V4 expression bead chip platform across the TUSC2-inducible H1299 clone [15, 16]. The datasets for the control and doxycycline-induced treatment groups were analyzed to generate specific sets of genes to identify differential expression levels between the control and doxycycline treatment groups. A volcano plot and a heat-map were used to visualize the results (Figure 3A and 3B). In the resulting gene profile that differentiated between the control and doxycycline treatment groups, most genes that were upregulated in the treatment group encoded proteins that inhibit the mTOR pathway (Figure 3C). Expression of these proteins was increased 3- to 4-fold (Figure 3C). Ingenuity pathway analysis showed the highest positive z-score for the cell cycle related pathways, which can be regulated via mTOR (Figure 3D). Cell cycle analysis using Flow Cytometry demonstrated TUSC2 induction caused a significant increase in S phase and a decrease in G1 phase (Supplementary Figure 2). These results indicate that *TUSC2* inhibits mTOR function indirectly through multiple pathways and proteins in NSCLC.

### TUSC2 prevents IFN-γ–induced PD-L1 expression

**Figure 4:**
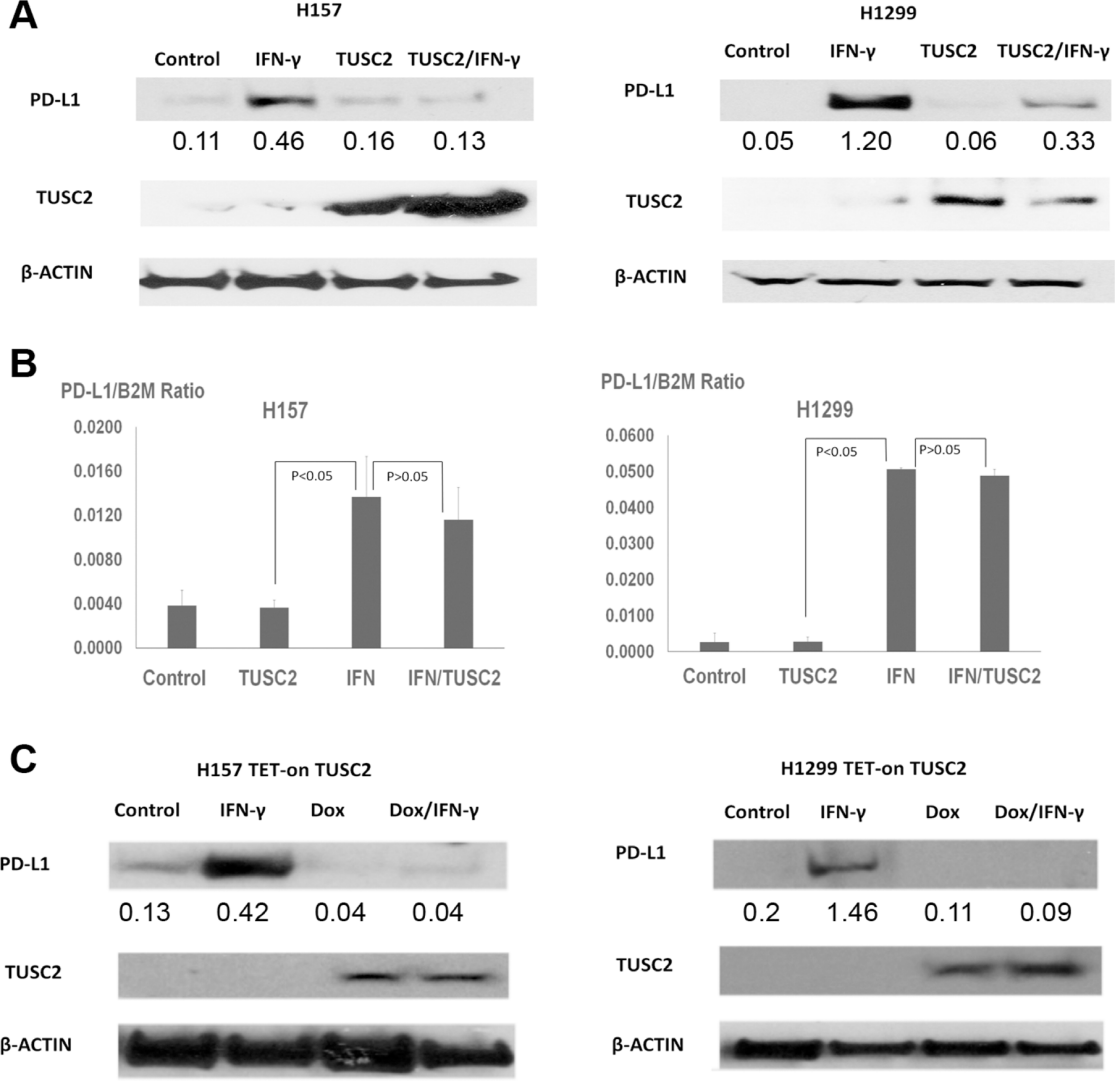
TUSC2 prevents IFN-γ–induced PD-L1 expression (**A**) H1299 and H157 cells were transiently transfected with 4 μg of TUSC2 cDNA, then treated with 10 ng/mL IFN-γ or control phosphate-buffered saline for 24 hours, harvested, and lysed. PD-L1, TUSC2, and β-actin control signals were then detected by Western blot analysis. These experiments were repeated twice. (**B**) H1299 and H157 cells were transiently transfected with 4 μg of TUSC2 cDNA, then treated with 10 ng/mL IFN-γ or control phosphate-buffered saline for 24 hours. PD-L1 transcriptions in these four groups were measured via quantitative real-time PCR. Data represent the means and standard deviations (error bars) of four separate experiments, statistically analyzed using a two-tailed *t*-test. (**C**) TUSC2-inducible H1299 and H157 cells were exposed to 1 μg/mL doxycycline (DOX), then exposed to 10 ng/mL IFN-γ or control phosphate-buffered saline for 24 hours, harvested, and lysed. PD-L1, TUSC2, and β-actin control signals were then detected by western blot analysis. These experiments were repeated twice. Expression of the targeted proteins was quantified by densitometry.

Once T cells infiltrate into tumor sites, many inflammatory cytokines are released upon antigen stimulation. Among these, IFN-γ is a strong PD-L1 stimulator [17]. Tumor cells then activate PD-L1 production, which in turn neutralizes T cell activity and causes T cell apoptosis. This offers tumors a vital survival mechanism to escape from immune toxicity. To investigate whether TUSC2 can prevent the upregulation of PD-L1 caused by IFN-γ, we transfected cancer cells with TUSC2 and subsequently exposed them to IFN-γ. Western blot analysis (Figure 4A) showed that IFN-γ can increase PD-L1 protein expression. The increase in PD-L1 expression was associated with increases in PD-L1 transcription (Figure 4B). Western blot analysis also demonstrated that TUSC2 expression could reduce PD-L1 expression in the presence of IFN-γ (Figure 4A). Furthermore, we deployed flow cytometry to quantify the PD-L1 reduction after TUSC2 transfection with or without IFN-γ. As demonstrated in Supplementary Figure 3, the TUSC2 expression could reduce PD-L1 expression with the presence of IFN-γ. To further investigate whether TUSC2 regulates PD-L1 at the transcriptional level, we measured PD-L1 mRNA levels in cancer cell lines after TUSC2 transfection with or without IFN-γ exposure. No statistically significant decrease (*p* > 0.05) in PD-L1 transcription was observed after TUSC2 transfection, whereas IFN-γ significantly increased PD-L1 transcription (*p* < 0.05) (Figure 4B). These results suggest that TUSC2 downregulated PD-L1 by inhibiting translation, instead of inhibiting transcription.

We performed a similar study using TUSC2-inducible H1299 and H157 cells (Figure 4C). Upon exposure to doxycycline, TUSC2 expression was detected by Western blot analysis in both cell lines. Subsequent IFN-γ exposure could induce PD-L1 expression. But increases in PD-L1 expression in both cell lines were diminished after TUSC2 induction. In addition, our microarray data for H1299-inducible cell lines showed that TUSC2 did not change PD-L1 transcription. These results further indicate that TUSC2 downregulates PD-L1 through translational, rather than transcriptional, mechanisms.

## DISCUSSION

Tumor response to immunotherapy is the outcome of crosstalk among tumor cells, cytokines, and immune-effector cells residing in the tumor microenvironment. A growing body of evidence indicates that oncogenes allow tumor cells to be autonomous by altering the immune system in the tumor microenvironment, in addition to playing a role in tumor apoptosis. Here, we showed that overexpression of TUSC2, a unique tumor suppressor [18], has the potential to sensitize NSCLC to T cell–dependent immunotherapy by altering the tumor microenvironment via PD-L1 downregulation.

A number of cellular stress conditions, such as nutrient deprivation, oxidative stress, hypoxia, and calcium flux disturbances, lead to metabolic stress, endoplasmic reticulum stress, and reduced protein translation. Our previous studies demonstrated that TUSC2 selectively inhibits mTOR activity and increases cellular reactive oxygen species production. These findings led to the hypothesis that TUSC2 could reduce some protein synthesis. In the current study, microarray analysis using TUSC2-inducible cell lines and expression profiling indicated that TUSC2 overexpression led to AKT-mTOR pathway inhibition in cancer cells, which was associated with decreased PD-L1 levels. Increased TUSC2 expression significantly enhanced the transcription of several genes, including *TRIB3*, *SESN2*, and *DDIT4*, which could negatively regulate AKT-mTOR pathway. Our data suggested such negative regulation mainly targeted the upstream kinases which could phosphorylate mTOR protein. TRIB3 is a negative regulator of NF-κB and can also negatively regulate the cell survival serine-threonine kinase AKT1 [16]. SESN2, a tumor suppressor, functions as an intracellular leucine sensor that negatively regulates the TORC1 signaling pathway through the GATOR complex [19, 20]. shRNA against SENS2 transfection was observed to increase mTOR activity in lung cancer [21]. *DDIT4* regulates cell growth, proliferation, and survival via inhibition of mTOR. Inhibition of mTOR is mediated by pathways that involve DDIT4/REDD1, AKT1, the TSC1-TSC2 complex, and the GTPase RHEB [22, 23]. Additionally, TUSC2 could regulate mTOR via AMPK which directly phosphorylates raptor (a component of mTORC1 complex) to inhibit mTOR signaling. As a myristoylated protein resides in the mitochondria, TUSC2 is found to regulate the re-programming of mitochondrial metabolism, intracellular Ca2+ distribution and ATP production, all of which were crucial for cells with high proliferative potential such as cancer cells and activated T lymphocytes. Overexpressed TUSC2 in cancer cells could decrease Ca2+ distribution, and ATP production. AMPK could then be activated in response to lower ATP/AMP ratio and in turn to inhibit mTOR activity. Taken together, these findings suggest that TUSC2 negatively regulates mTOR activity through a network of proteins that converge on the mTOR pathway.

Our data provided a direct link between mTOR activity and inhibition of PD-L1, which could be upregulated after IFN-γ stimulation. The regulation of PD-L1 is complex and most likely depends on the status of underlying transcriptional and signaling networks as well as protein translation, in a mechanism parallel to but independent of activation of the JAK/STAT pathway or HIF1 pathway. In the current study, we showed that treatment with an mTOR inhibitor could decrease PD-L1 protein expression in NSCLC cells. Although PD-L1 transcription does not depend on mTOR activation, translation of IFN-γ–induced PD-L1 transcripts may be dependent on activation of PI3K, AKT, and mTOR kinase activity.

A similar dependence of PD-L1 translation on PI3K/AKT/mTOR activity was also observed during viral infections. In HIV-1–infected cells, the viral protein Nef induced PD-L1 transcription by binding to the promoter, but PD-L1 protein expression depended on active PI3K/AKT signaling residing within the HIV-infected cells [24]. PD-L1 protein induction could be observed only in the virus-infected cells with high PI3K/AKT/mTOR activity. PD-L1 remained at low levels in cells with low mTOR activity. In the current study, we showed that inhibition of mTOR activity via TUSC2 expression could downregulate PD-L1 expression at the translational level even in the presence of strong IFN-γ stimulation. After TUSC2 transfection, PD-L1 production was inhibited after IFN-γ induction. Our study thus further demonstrated that TUSC2 transfection could prevent IFN-γ–induced PD-L1 expression. This suggests that TUSC2 overexpression–induced mTOR inhibition was necessary for PD-L1 downregulation.

IFN-γ is one of the central cytokines that coordinate tumor immune responses and the associated biological consequences. Upon activation, tumor-infiltrating lymphocytes residing in the tumor microenvironment produce IFN-γ which initiates cell cycle arrest and induces apoptosis in adjacent tumor cells. IFN-γ shows antitumor activity in patients with advanced head and neck squamous cell carcinoma and NSCLC [25]. Unfortunately, tumor cells develop escape mechanisms from the IFN-γ–mediated immune responses by inducing PD-L1 overexpression. Increased PD-L1 expression induces apoptosis in tumor-infiltrating lymphocytes and then subsequently reduces the IFN-γ production through a negative feedback loop. This mechanism suggests that using rapamycin in combination with a PD-L1–blocking antibody could increase antitumor immunity. Although multiple trials of rapamycin or rapamycin analogues in cancer patients have shown no evidence of immunosuppression, implementing rapamycin in the immunotherapy context should be done with caution. This is because rapamycin has a black box warning from the US Food and Drug Administration stemming from concerns about the role of rapamycin in immunosuppression in patients undergoing renal transplantation who were also taking cyclosporine and corticosteroids raising issues about its role in immunosuppression [26, 27].

Studies have shown that some aspects of T cell activation are dependent on mTOR activity [28]. A nanoparticle TUSC2 delivery system, can achieve selective TUSC2 overexpression in NSCLC cells while sparing T cell and B cell populations. This unique feature of nanoparticle TUSC2 offers some advantage in designing a immunotherapy drug combinations.

Study of TUSC2-modified lung cancer cells may reveal novel vulnerabilities targetable by existing immunotherapies. A clinical trial showed that the *TUSC2* gene could be delivered intravenously in patients using nanoparticles to achieve sufficient TUSC2 protein expression levels in tumors to mediate a clinical response. Thus, the combination of TUSC2 with T cell–based immunotherapies is feasible in future clinical trials for patients.

## MATERIALS AND METHODS

### Cell culture

Human NSCLC cell lines with various TUSC2 expression, HCC827, H1299, H1975, and H157, were provided by Drs. John V. Heymach (MD Anderson) and Adi Gazdar and John D. Minna (The University of Texas Southwestern Medical Center at Dallas). Cells were maintained in RPMI-1640 medium (Manassas, VA) supplemented with 10% heat-inactivated fetal bovine serum (Invitrogen, Carlsbad, CA).

### Reagents

IFN-γ, Doxycycline and rapamycin were purchased from Sigma-Aldrich Corporation (Saint Louis, MO). Antibodies to detect mTOR, phospho-mTOR, p70 S6 kinase, phospho-p70 S6 kinase, S6 ribosomal protein, and phospho-S6 ribosomal protein were purchased from Cell Signaling (Danvers, MA). PD-L1 antibody was purchased from Abcam (Cambridge MA). The TUSC2 polyclonal antibody was developed in Bethyl Laboratories (Montgomery, TX). Lipofectamine 2000 was purchased from Invitrogen. Nanoparticle–TUSC2 complexes were made as previously described [29].

### Stable Tet-inducible TUSC2 cell lines

Tet-inducible TUSC2-expressing H1299 and H157 cells were produced using the Lenti-X Tet-On advanced inducible expression system (Clontech, Mountain View, CA) as described previously. TUSC2-induced H1299 and H157 cells were selected after exposure to 2 μg/mL doxycycline for 48 hours and assessed for TUSC2 expression using Western blot analysis.

### Western blotting analysis

Cancer cells were lysed using RIPA lysis buffer, and equal amounts of total proteins were resolved on 4–20% Tris-glycine gels and transferred onto a nitrocellulose membrane. The membranes were then incubated with a rabbit anti-human PD-L1 polyclonal antibody, a rabbit anti-human mTOR polyclonal antibody, a rabbit anti-human phospho-mTOR polyclonal antibody, rabbit anti-human phospho-S6 ribosomal protein polyclonal antibody, rabbit anti-human total S6 ribosomal protein polyclonal antibody, rabbit anti-human phospho-p70 S6 kinase polyclonal antibody, rabbit anti-human total p70 S6 kinase polyclonal antibody, rabbit anti-human TUSC2 polyclonal antibody and a mouse anti-human β-actin antibody overnight. Antibody binding was then detected using chemiluminescence (Cell Signaling Technology, Danvers, MA) and signals were visualized by autoradiography.

### Quantitative real-time RT-PCR

To determine the expression of PD-L1 mRNA), total RNA was extracted using TRIzol Reagent^®^ (Life Technologies) according to the manufacturer's protocol. Equal amounts of RNA were reverse transcribed using a High-Capacity cDNA Reverse Transcription Kit (Applied Biosystems, Foster City, CA). cDNAs were then amplified by real-time PCR using TaqMan^®^ gene expression assays (Applied Biosystems) for PD-L1 (Product ID: Hs01125301). The housekeeping B2M gene was used as internal controls.

### Illumina gene expression

Total RNAs extracted from Tet-inducible TUSC2 H1299 cells were reversed-transcribed to generate amplified biotinylated cRNA, then hybridized overnight to Illumina HT-12 bead arrays, washed, and stained with streptavidin-Cy3. Arrays were scanned on a bead array reader (Illumina). Raw measurements of the intensity of each bead were captured directly and processed as “bead-level” data. All measurements were processed as “probe-level” data by GenomeStudio software (Illumina). One-way analysis of variance (ANOVA) was used to identify the differentially expressed genes. Benjamini-Hochberg method was used to adjust for multiple hypothesis testing and estimate false discovery rate (FDR) [30]. FDR q value cutoff of 0.002 (corresponding *p* value = 0.00025) was used to identify significant genes by one-way ANOVA. Tukey honest significant difference tests were used to do the *post hoc* pairwise comparisons. Each group had three replicates. Genes with *p* < 0.05 by Tukey tests and fold changes ≥2 or ≤–2 were considered significantly differentially expressed. A volcano plot and a heatmap for each comparison were generated. In the heatmap, two-way clustering with Pearson distance metric and Ward's minimum variance method was used to illustrate the expression profile of the identified significant genes. Ingenuity pathway analysis tool (http://www.ingenuity.com) was used to determine which pathways were differentially represented in the identified significant genes, compared to the Ingenuity knowledge base.

### Statistical analysis

The statistical analysis for gene expression data was performed using R packages (https://cran.r-project.org/web/packages/; and https://www.bioconductor.org/). Other statistical analysis was performed using SAS 9.4 (SAS Institute Inc., Cary, NC). All data were presented as mean ± standard deviation. The statistical significance of differences between treatments was tested using three-way analysis of variance and two-tailed *t*-tests; *p* < 0.05 was considered significant.

## SUPPLEMENTARY MATERIALS FIGURES


