# Supplementary material for: TUSC2 downregulates PD-L1 expression in non-small cell lung cancer (NSCLC)
Source: Oncotarget. 2017 Nov 21;8(64):107621–9. doi: 10.18632/oncotarget.22581 (PMC5746095; doi:10.18632/oncotarget.22581)
Supplement: Supplementary file 1 [file oncotarget-08-107621-s001.pdf]

TUSC2 downregulates PD-L1 expression in non-small cell lung cancer (NSCLC)

SUPPLEMENTARY MATERIALS

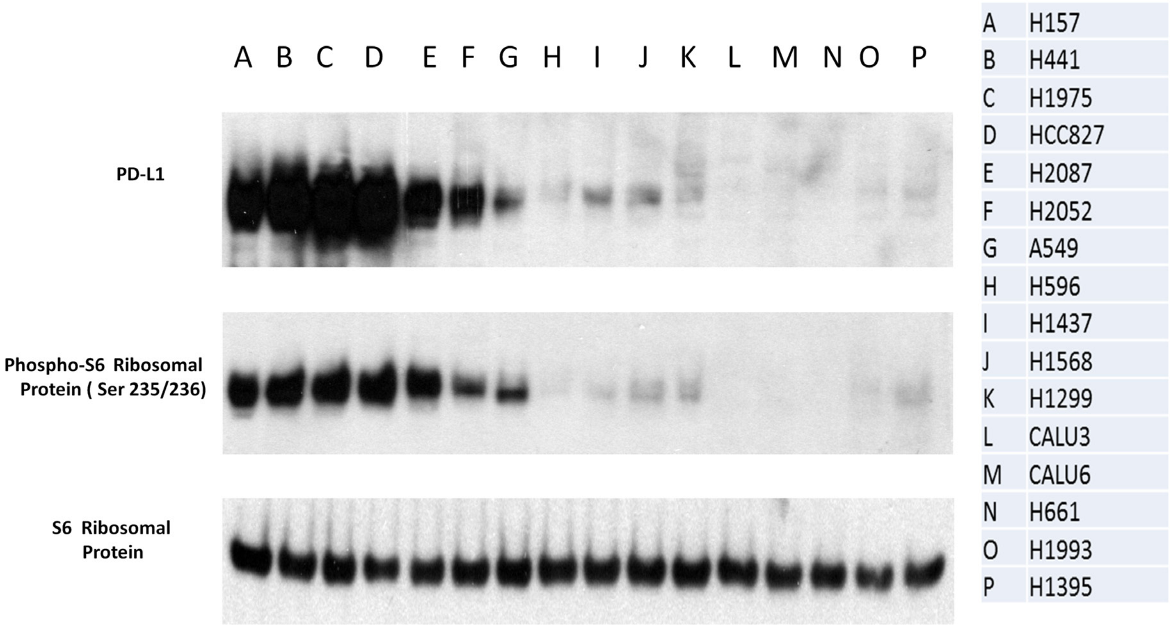

Supplementary Figure 1: Baseline expression of PD-L1 and mTOR activities. PD-L1 expressions in sixteen lung cancer cell lines, including H157, H1975, and HCC827, were evaluated by western blotting using anti-PD-L1 antibody. Among these cell lines, mTOR activities were measured via anti- Phospho-S6 Ribosomal Protein (Ser 235/236) western blotting. S6 Ribosomal protein was used as loading control.

PBS control

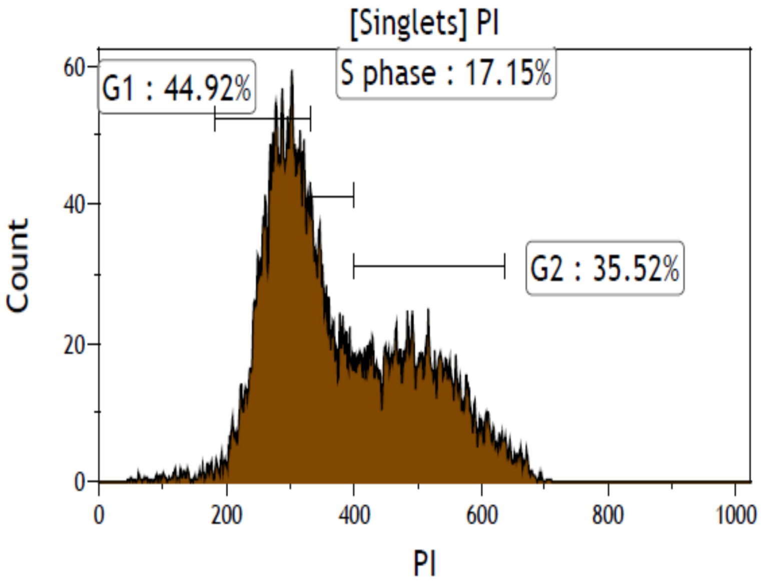

TUSC2 induction via Doxycycline

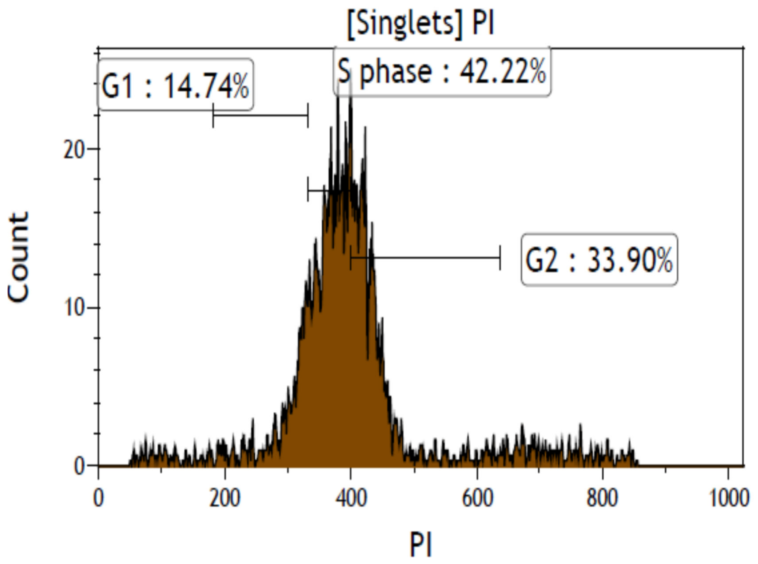

Supplementary Figure 2: TUSC2 induction led to cell cycle arrest. Tet-inducible TUSC2 H1299 clones exposed to 1 µg/mL doxycycline or PBS control for 48 hours and then stained with propidium iodide. The DNA content was analyzed by flow cytometry.

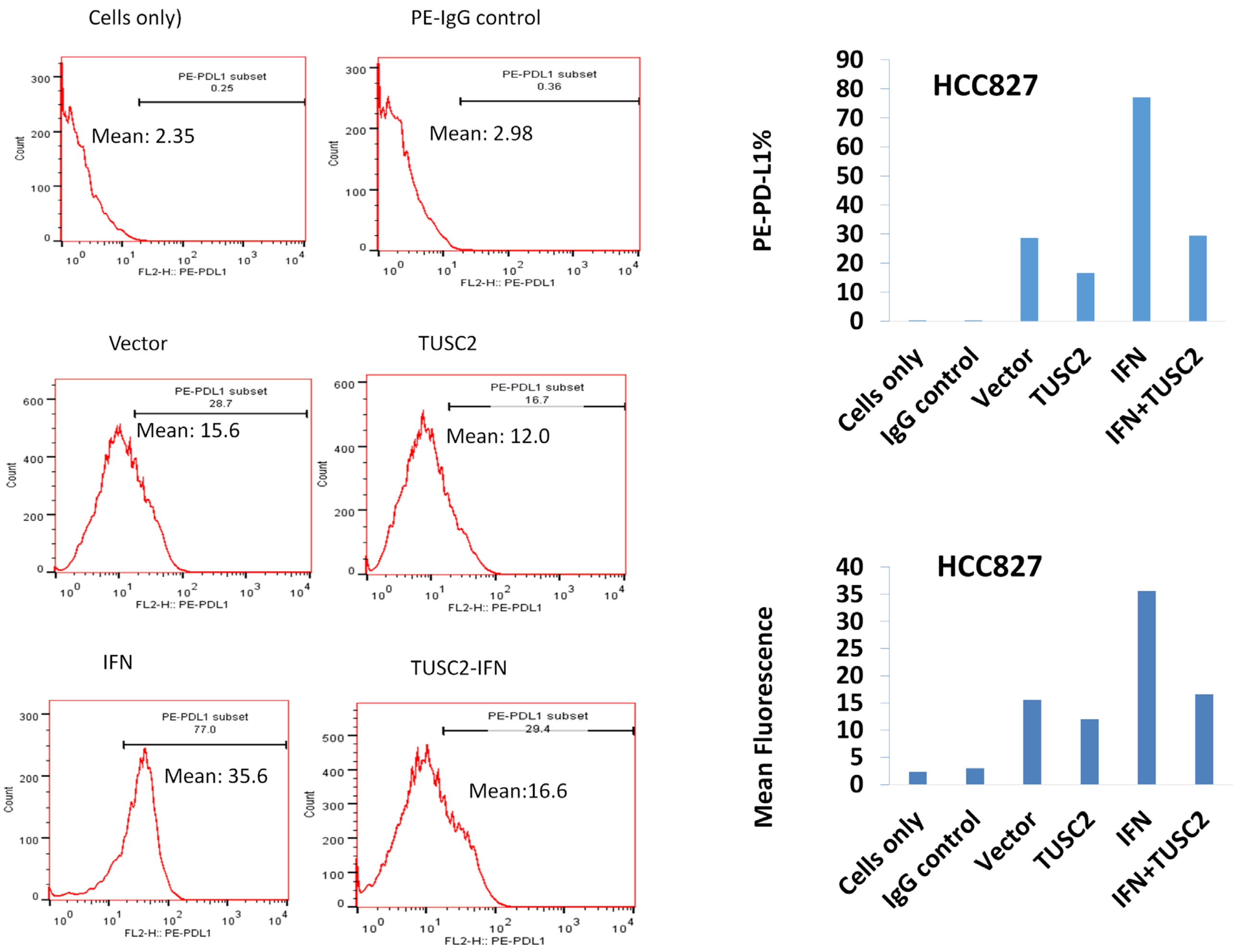

Supplementary Figure 3: Flow cytometry analysis of PDL-L1 expression in HCC827 cells with or without TUSC2 transfection and IFN-γ. HCC827 cells were transiently transfected with 4 µg of TUSC2 cDNA, then treated with 10 ng/mL IFN-γ or control phosphate-buffered saline for 24 hours. Cell surface PD-L1 in these groups were measured via flow cytometry.
